# An Improved Binary Walrus Optimizer with Golden Sine Disturbance and Population Regeneration Mechanism to Solve Feature Selection Problems

**DOI:** 10.3390/biomimetics9080501

**Published:** 2024-08-18

**Authors:** Yanyu Geng, Ying Li, Chunyan Deng

**Affiliations:** 1 College of Computer Science and Technology, Jilin University, Changchun 130012, China; 2 Key Laboratory of Symbolic Computation and Knowledge Engineering of Ministry of Education, Jilin University, Changchun 130012, China

**Keywords:** metaheuristic optimization, feature selection, walrus optimizer, chaos mapping, population regeneration mechanism

## Abstract

Feature selection (FS) is a significant dimensionality reduction technique in machine learning and data mining that is adept at managing high-dimensional data efficiently and enhancing model performance. Metaheuristic algorithms have become one of the most promising solutions in FS owing to their powerful search capabilities as well as their performance. In this paper, the novel improved binary walrus optimizer (WO) algorithm utilizing the golden sine strategy, elite opposition-based learning (EOBL), and population regeneration mechanism (BGEPWO) is proposed for FS. First, the population is initialized using an iterative chaotic map with infinite collapses (ICMIC) chaotic map to improve the diversity. Second, a safe signal is obtained by introducing an adaptive operator to enhance the stability of the WO and optimize the trade-off between exploration and exploitation of the algorithm. Third, BGEPWO innovatively designs a population regeneration mechanism to continuously eliminate hopeless individuals and generate new promising ones, which keeps the population moving toward the optimal solution and accelerates the convergence process. Fourth, EOBL is used to guide the escape behavior of the walrus to expand the search range. Finally, the golden sine strategy is utilized for perturbing the population in the late iteration to improve the algorithm’s capacity to evade local optima. The BGEPWO algorithm underwent evaluation on 21 datasets of different sizes and was compared with the BWO algorithm and 10 other representative optimization algorithms. The experimental results demonstrate that BGEPWO outperforms these competing algorithms in terms of fitness value, number of selected features, and *F*1-*score* in most datasets. The proposed algorithm achieves higher accuracy, better feature reduction ability, and stronger convergence by increasing population diversity, continuously balancing exploration and exploitation processes and effectively escaping local optimal traps.

## 1. Introduction

With the exponential growth and evolution of IT and Internet applications [1] in the past few years, there has been an unprecedented expansion in the magnitude and dimensions of data in a range of disciplines [2]. It has brought challenges in the realms of data mining and machine learning.

Extensive and intricate data encompass a significant volume of valuable information, as well as redundant and irrelevant information [3], which increases computational and storage costs [4,5], reduces classification performance and efficiency [6], and decreases data performance [7]. This calls for a solution that can reduce the dimensions of the original data. Dimensionality reduction is an effective method that can reduce data dimensions and computational complexity, reduce storage space, and establish a more generalized model [8].

FS, one of the most critical strategies for dimensionality reduction, effectively reduces storage and computing costs by eliminating irrelevant and repetitive features, while preserving the physical significance of the initial characteristics and endowing the model with better legibility and intelligibility [9,10,11]. The FS technique plays an essential part in the preparation of data for subsequent tasks (e.g., classification) by analyzing the most relevant features [12,13]. It has been extensively utilized in various areas, including image processing [14,15], text mining [16], social and behavioral science [17], biomedical research [18,19,20], fault diagnosis [21], and so on.

According to evaluation criteria, typically, FS methods can be divided into three different groups: filter, wrapper, and embedded models [22]. The filter chooses features based on their correlation with latent variables without the influence of any specific learning algorithm [23]. The wrapper evaluates each feature subset using training models such as KNN and SVM to acquire the best subset [24]. The embedded approach incorporates the process of FS within the training model and uses a specific structure to guide feature selection. Wrappers are frequently employed for addressing issues related to FS because they are better than filters in terms of the precision of classification and have a wider range of applications than embedded methods [25].

The wrapper method repeatedly searches for feature subsets and evaluates the selected features until it reaches the stopping criterion [7]. In wrapper-based FS methods, feature subsets’ quality is evaluated using support vector machine (SVM), decision tree (DT), artificial neural network (ANN), and k-nearest neighbor (KNN) as fitness functions searching for feature subsets is the NP-hard problem [26]. The simplest approach to searching is to examine every potential combination of features, a process referred to as the exponential algorithm [27], but its computational cost is extremely high and it is practically impossible to apply [28]. In order to reduce computational costs, sequential algorithms have been suggested, which select or delete features in the design order [29,30]. However, once a certain feature is selected or deleted, it cannot be manipulated again, which leads to a local optimum. Over the past few years, random search algorithms have gained more attention for their advantage of exploring the randomness of feature space and effectively preventing the algorithm from getting stuck in a suboptimal solution within a specific region. Metaheuristic, the most promising solution in random search algorithms, has become one of the suitable solutions for FS owing to its excellent performance demonstrated in various optimization scenarios [31].

There are four main sources of inspiration for metaheuristics: evolutionary-based, physics-based, human-based, and swarm intelligence-based FS algorithms [32]. SI algorithms have been shown to be competitive with the other three algorithms mentioned above due to fewer parameters, faster convergence speed, better equilibrium between exploring and exploiting, and better performance [33,34]. The widely recognized representative algorithms of SI algorithms are particle swarm optimization (PSO) [35], salp swarm algorithm (SSA) [36], whale optimization algorithm (WOA) [37], etc.

Although swarm intelligence algorithms are effective in solving FS problems, they still face problems such as stagnation of local minimum, premature convergence, unbalanced exploration and exploitation, and low population diversity. To enhance the efficiency of algorithms based on SI in FS, there is a need to seek an algorithm that can deal with the above challenges.

The walrus optimizer (WO) algorithm, introduced by Han et al., is a new SI algorithm that takes inspiration from the conduct of walrus groups. WO can be a desirable option due to its capacity for adaptation, minimal parameters, and powerful mechanisms for balancing exploration and exploitation. The effectiveness of the WO algorithm in addressing continuous issues has been demonstrated through experimental research [38,39,40]. However, the WO algorithm still suffers from low population diversity, slow convergence, and inability to fully utilize the problem domain. In addition, the traditional WO algorithm was created to deal with continuous problems, and there has been no research or design directed at using WO for FS. This situation motivated us to enhance the original WO algorithm and design its binary version for FS tasks.

This paper aims to investigate an optimization algorithm that can improve population diversity and overcome local optimal stagnation while providing high optimization performance. In this research, initially, a refined WO algorithm, BGEPWO, is introduced, which uses an ICMIC chaotic map to initialize the population, introduces an adaptive operator to update the safety signal, and proposes a population regeneration mechanism to eliminate old and weak individuals while generating promising new ones. In addition, the EOBL strategy and golden sine strategy are used, where EOBL is used to update the escape direction of the walrus, and golden sine is used to perturb the population. The proposed algorithm improves population diversity through various mechanisms, continuously balances exploration and exploitation during the optimization process, avoids falling into local optima, and accelerates convergence speed, thereby improving the performance of the algorithm. Then, a binary version BGEPWO algorithm was developed. 21 datasets from the UCI and ASU have been chosen to assess the effectiveness of BGEPWO, and conducted a comparison with BWO, and other 10 metaheuristic algorithms, including binary artificial bee colony algorithm (BABC) [41], binary particle swarm optimization algorithm (BPSO), binary bat algorithm (BBA) [42], binary whale optimization algorithm (BWOA), binary Kepler optimization algorithm (BKOA) [43], binary salp swarm algorithm (BSSA), binary Nutcracker optimizer algorithm (BNOA) [44], binary Harris hawks optimization (BHHO) [45], binary crested porcupine optimizer (BCPO) [46], and binary coati optimization algorithm (BCOA) [47].

The experimental findings indicate that the improved BGEPWO significantly enhances the ability of the WO algorithm to solve FS problems. In most cases, the BGEPWO outperforms the WO algorithm and competing algorithms in terms of fitness value, number of selected features, and *F*1-*score*. In addition, the use of a 5% Wilcoxon rank-sum test validated that BGEPWO performs significantly better than competitive algorithms on most datasets.

The main contributions of this research can be succinctly delineated as follows:

- Initializing the population using ICMIC chaotic mapping instead of the random way in the original algorithm is able to improve variety in the population and prevent early convergence.
- BGEPWO employs a new adaptive safety signal, enhancing the algorithm’s stability and promoting its convergence through the introduction of the adaptive operator.
- The population regeneration mechanism is adopted to improve the development competence by eliminating old and weak individuals and generating promising new ones, so as to facilitate the ongoing movement of the population towards the best solution and accelerate the convergence process.
- The EOBL strategy is employed in the escape behavior of walruses, which enables the method to flee from the current local optimum bottleneck while expanding the search range and improving the diversity.
- This proposed method employs the golden sine strategy to perturb the walrus population in the advanced phase of each iteration, enabling the method to explore the search area more thoroughly during the iteration process, enhancing the algorithm’s capacity for exploration, effectively solving the issue of settling into local traps and thus accelerating convergence speed.

The remaining sections of this paper are structured in the following manner: Section 2 presents the relevant studies regarding the utilization of metaheuristic algorithms in the field of FS. The WO algorithm is presented in Section 3. It offers a detailed introduction of the proposed BGEPWO in Section 4. Section 5 describes the experimental design and result analysis. Section 6 provides a summary of the paper and offers potential directions for future research.

## 2. Related Works

Feature selection methods typically search for feature subsets within the solution domain, which is an NP-hard problem. Metaheuristic algorithms provide an effective approach for addressing intricate optimization and NP-hard problems, enabling the discovery of acceptable solutions within a reasonable timeframe [26]. Metaheuristic algorithms can be divided into evolutionary-based, physics-based, human-based, and swarm intelligence-based feature selection algorithms.

In order to implement feature selection using these algorithms, continuous search space is usually mapped to feature space by means of transfer functions, logical operators, etc. [48]. Over recent years, an increasing pattern has been observed in the utilization of metaheuristic algorithms by researchers to address the process of selecting features in various fields. The related work is elaborated in this section.

Evolutionary algorithms take inspiration from the mechanism of natural progression and use operations on the best solution to create new individuals. Common evolutionary-based algorithms encompass genetic algorithms (GAs) [49], differential evolution (DE) [50], etc. To tackle the challenges of avoiding local pitfalls and reducing computational expenses, Tarkhaneh et al. [51] proposed an improved MDEFS method using two novel mutation methodologies and demonstrated its superiority. Maleki et al. [52] combined a classic genetic algorithm with a KNN classifier to effectively downscale the dimensionality of patient disease datasets and enhance the precision of disease identification. FSGAS is a GA-based FS method studied by Berrhail et al. [53] for identifying the crucial and pertinent features of compounds in ligand-based virtual screening (LBVS), which effectively improves screening performance.

Methods derived from physics are derived from physical rules in the universe [54], mainly including simulated annealing (SA) [55], gravitational search algorithm (GSA) [56], and so on. On the basis of extracting candidate lesions features of diabetes retinopathy (DR), Sreng et al. [57] used hybrid simulated annealing (SA) for feature selection to enable automated DR screening. Albashish et al. [58] combined the proposed model based on binary biogeography optimization (BBO) with SVM to achieve better accuracy than the BBO methods and other extant algorithms. Taradeh et al. [59] added evolutionary crossover and mutation operators to the GSA to implement FS. Comparison experiments with GA, PSO, and GWO using KNN and DT as classifiers unfolded on the UCI datasets have demonstrated its superiority in dealing with FS problems. To improve the efficiency of virtual screening (VS) in drug discovery campaigns, Mostafa et al. [60] recommended an FS framework consisting of a gradient-based optimizer (GBO) and KNN. The effectiveness of the suggested research on the high-dimensional dataset and the low-dimensional dataset is validated on real-world benchmark datasets. Dong et al. [19] improved the dandelion algorithm (DA) using the sine cosine operator, restart strategy, and quick bit mutation. The suggested SCRBDA algorithm was evaluated against eight other classical FS algorithms on the UCI datasets. The outcomes demonstrated the excellent feature reduction ability and performance of SCRBDA.

Algorithms based on human behavior take inspiration from the actions and patterns of humans, in which each person has a way of influencing the behavior of the group. Some common human-based algorithms are the teaching–learning-based optimization (TLBO) algorithm [61], brainstorm optimization (BSO) [62], etc. For the purpose of augmenting the exploratory prowess in BSO, Oliva et al. [63] improved it using chaotic mapping, opposition-based learning, and disruption operators. The new algorithm effectively improved the efficiency of FS by enhancing the population’s variety. Manonmani et al. [64] utilized the enhanced TLBO for the classification and projection of chronic kidney disease (CKD), reducing the quantity of features needed for diagnosing CKD. To increase the variety in the search procedure and handle the duality of FS problems, Awadallah et al. [65] developed the BJAM algorithm using adaptive mutation rate and sinusoidal transfer function and validated the suggested algorithm’s efficiency by employing KNN as a classifier on 22 datasets. Based on the gaining–sharing knowledge-based optimization algorithm (GSK), Agrawal et al. [66] proposed a binary version named FS-NBGSK and demonstrated its excellent performance in terms of accuracy, convergence, and robustness on the benchmark datasets using the KNN classifier. In the study of Xu et al. [67], Six distinct transfer functions were employed to produce six binary editions of the arithmetic optimization algorithm (AOA), and then the integrated transfer function and Lévy flight were employed to optimize the search performance, and the BAOA_S1LF algorithm demonstrated its superiority among the six methods tested on the UCI datasets.

The algorithm that makes use of swarm intelligence (SI) is inspired by the combined actions of a group of animals residing in communities [68]. In swarm intelligence algorithms, individuals share their exploration of the search domain, making the whole group continuously move towards a better solution and ultimately approach the optimal solution [69].

In order to identify effective features in chemical compound datasets, Houssein et al. [70] combined Harris hawk optimization with SVM and KNN to formulate two classification algorithms, HHO-SVM and HHO-KNN. Experiments on chemical datasets of monoamine oxidase and QSAR biodegradation showed that the former method achieved the highest optimization feature ability among a group of competing algorithms.

In the newly proffered multi-population-based PSO method, Kılıç et al. [71] utilized stochastic and relief-based methods for initialization and used two populations for simultaneous search. Their experimental results, based on 29 datasets, demonstrate that the mean accuracy in classifying of MPPSO consistently surpassed that of other algorithms.

Wang et al. [72] suggested a hybrid sine–cosine chimp optimization algorithm (ChOA), which utilizes a multiloop iteration approach to enhance the integration of the sine–cosine algorithm (SCA) and ChOA and performs binary transformation through an S-shaped transfer function. Experiments using the KNN classifier on 16 datasets have shown that this approach demonstrates outstanding efficacy in mitigating FS challenges, surpassing other algorithms in performance.

In an effort to minimize redundant characteristics and enhance classification accuracy, Shen et al. [73] introduced a refined fireworks algorithm (FA) by designing an innovative fitness evaluation method and a fitness-based roulette wheel selection strategy, while introducing a differential mutation operator. The effectiveness of this strategy and the importance of joint optimization were validated through experiments on 14 UCI datasets.

Tubishat et al. [74] proposed the IWOA algorithm by integrating elite opposition-based learning (EOBL) and evolutionary operators with the whale optimization algorithm (WOA). Sentiment analysis was performed on four Arabic benchmark datasets using SVM classifiers. The experimental findings evidenced the superior efficacy of the novel algorithm in comparison with the alternative algorithm on the indicators of precision in categorization and minimization of feature subset size to the greatest extent possible.

For the purpose of analyzing and addressing FS issues in biological data, Seyyedabbasi et al. [75] recommended the binary version of the sand cat swarm optimization (SCSO) algorithm, bSCSO. The evaluation on the dataset established the superiority of the bSCSO algorithm in high predictive performance and small feature size.

In the newly proposed adaptive ranking moth-flame optimization (ARMFO) algorithm, Yu et al. [76] used ranking probability, adaptive chaotic mutation, and greedy selection to improve the search capability of the algorithm, then achieved satisfactory outcomes on the UCI datasets.

However, the metaheuristic algorithms described above have some limitations when dealing with the feature selection problem. First, the algorithms used in some studies have more parameters, and the selection of parameters has a significant impact on the performance as well as computational cost of the algorithms. Second, some algorithms have an imbalance between exploration and exploitation in the search process, which leads to a decrease in search performance and convergence depth. Third, many studies only focus on a single problem, and the generalized performance of the algorithms fails to be verified. Finally, some studies have limitations in the dimensionality of the dataset chosen for feature selection, and there is no extensive discussion on the data dimensions from low to high. To address the above issues, this paper developed the BGEPWO algorithm for feature selection on datasets of different dimensions, aiming to improve the algorithm’s performance, computational efficiency, robustness, diversity of solutions, and balance of search strategies.

## 3. Walrus Optimizer

Walruses are large group-living amphibious mammals. Walrus herds are composed of male walruses, female walruses, and juvenile walruses. They generally live in deep water, rely on sound to locate and transmit signals, and cooperate in foraging. In a herd of walruses, there are two walruses acting as vigilantes. They are alert to the surrounding situation and transmit signals to their companions. When encountering enemies, the herd of walruses adopts a collective defense mechanism for self-protection [38,39,40,41,42,43,44,45,46,47,48]. During the breeding season, male walruses establish territories on land to attract female walruses for reproduction.

The primitive WO algorithm is derived from the collective behavior of walrus groups, and mathematically simulated the walruses’ behavior of migration, reproduction, roosting, foraging, gathering, or escape after receiving signals [38]. This section elaborates on the mathematical modeling of the original WO algorithm.

### 3.1. Walrus Optimizer

In the walrus optimization algorithm, the optimization process starts from initialization, which generates random candidate solution X in the solution space, as follows:

(1) X=LB+rand(UB−LB)

where UB and LB constitute the problem’s upper and lower limits. rand is a uniform random vector within (0,1).

X represents the WO population matrix, as shown in Equation (2), where the individuals are the agents during the process of optimizing, and they regularly update their positions during the iteration process. F represents the fitness function values corresponding to each agent, as shown in Equation (3).

(2) $$X={\left[\begin{array}{c} X_{1}\\ \vdots \\ X_{i}\\ \vdots \\ X_{n} \end{array}\right]}_{n\times d}={\left[\begin{array}{ccccc} X_{1,1} & \cdots & X_{1,j} & \cdots & X_{1,d}\\ \vdots & \ddots & \vdots & & \vdots \\ X_{i,1} & \cdots & X_{i,j} & \cdots & X_{i,d}\\ \vdots & & \vdots & \ddots & \vdots \\ X_{n,1} & \cdots & X_{n,j} & \cdots & X_{n,d} \end{array}\right]}_{n\times d}$$

(3) $$F={\left[\begin{array}{c} F_{1}\\ \vdots \\ F_{i}\\ \vdots \\ F_{n} \end{array}\right]}_{n\times d}={\left[\begin{array}{ccccc} (f_{1,1} & \cdots & f_{1,j} & \cdots & f_{1,d})\\ \vdots & \ddots & \vdots & & \vdots \\ (f_{i,1} & \cdots & f_{i,j} & \cdots & f_{i,d})\\ \vdots & & \vdots & \ddots & \vdots \\ (f_{n,1} & \cdots & f_{n,j} & \cdots & f_{n,d}) \end{array}\right]}_{n\times d}$$

where n represents the population size, d represents the dimension, $X_{i,j}$ represents the position of *i*th agent on *j*th dimension.

### 3.2. Signal Simulation

Walrus groups remain vigilant during foraging and roosting, with two walruses patrolling as vigilant individuals. When an unexpected situation is detected, they send signals to alert the walrus group. The definition of danger signal DangerSig and safety signal SafetySig in the behavior of walruses is as Equations (4) and (8).

(4) DangerSig=A×R

(5) A=2×α

(6) $$\alpha =1-\frac{t}{T}$$

(7) $$R=2\times r_{1}-1$$

(8) $$SafetySig=r_{2}$$

where A and R are risk factors that exhibit a linearly decreasing trend from 1 to 0 over the course of the iteration process. t signifies the current iteration count, and T signifies the maximum iteration count. $r_{1}$ and $r_{2}$ are random numbers fall within a specific range of (0,1).

### 3.3. Migration (Exploration)

The alert signals received by the herd of walruses from their peers are the key influence on the “action plan”. When the risk factors are too high (high danger signal values), the walrus group migrates to other areas (i.e., new domains in the solution space). When the risk factors are not as high (low danger signal values), the herd of walruses breeds in the ocean currents.

For the safety and survival of the population, when the level of risk becomes excessive, the walrus herd relocates to areas that are better suited for the survival of the herd. During migration, the location of each walrus is revised according to Equation (9).

(9) $$X_{i,j}^{t+1}=X_{i,j}^{t}+MigrationStep$$

(10) $$MigrationStep=(X_{m}^{t}-X_{n}^{t})\times \beta \times r_{3}^{2}$$

(11) $$\beta =1-\frac{1}{1+\exp(-\frac{t-T/2}{T}\times 10)}$$

where $X_{i,j}^{t}$ is the current position of the *i*th walrus on the *j*th dimension, and $X_{i,j}^{t+1}$ represents the updated location of the *i*th walrus on the *j*th dimension. MigrationStep is the step size of walrus movement. $X_{m}^{t}$ and $X_{n}^{t}$ represent the locations of two vigilantes chosen at random from the herd at the current iteration, respectively. The control factor of the migration step size β changes into a smooth curve with iteration. $r_{3}$ is within (0,1).

### 3.4. Reproduction (Exploitation)

When the level of risk factors is minimal, walruses reproduce, and during reproduction, the walrus group engages in two main behaviors: roosting on land and foraging underwater. The safety signal plays a crucial role in this process, influencing whether walruses choose roosting or foraging behavior.

#### 3.4.1. Roosting Behavior

Walrus herds choose to roost when the safety signal value is high. The walrus herd consists of male walruses, female walruses, and juvenile walruses, with a general proportion of 45%, 45%, and 10% in the population, respectively. In addition, the ratio of male to female walruses is basically maintained at 1:1. During the roosting process, different agent roles use different methods to update their positions.

1. Redistribution of male walruses

In order to enhance the variety of the populace and broaden the scope of research, the Halton sequence distribution was used to randomly distribute the positions of male agents.

2. Position update of female walruses

In the herd, male and leader walruses have an impact on the behavior of female walruses. The mathematical model in Equation (12) demonstrates the simulation of updating the female walrus’s location.

(12) $$Female_{i,j}^{t+1}=Female_{i,j}^{t}+\alpha \times (Male_{i,j}^{t}-Female_{i,j}^{t})+(1-\alpha )\times (X_{best}^{t}-Female_{i,j}^{t})$$

where $Female_{i,j}^{t}$ signifies the present location of the *i*th female agent on the *j*th dimension, $Female_{i,j}^{t+1}$ signifies the updated new location of the *i*th female walrus on the *j*th dimension, and $Male_{i,j}^{t}$ signifies the current location of the *i*th male agent on the *j*th dimension. $X_{best}^{t}$ signifies the leader walrus, which is the most efficient solution within the current iteration.

3. Position update of juvenile walruses

Young agents are frequently hunted by natural enemies because of their weakness. To avoid predation, juvenile agents should revise their present location in the following manner:

(13) $$Juvenile_{i,j}^{t+1}=P\times (S^{t}-Juvenile_{i,j}^{t})$$

(14) $$S^{t}=X_{best}^{t}+Juvenile_{i,j}^{t}\times LF$$

(15) $$LF=L\text{é}vy(\lambda )=0.05\times \frac{x}{{\left|y\right|}^{\frac{1}{\lambda }}}$$

(16) $$\sigma_{x}={\left[\frac{\Gamma (1+\varepsilon )\sin(\frac{\varepsilon \pi }{2})}{\Gamma (\frac{1+\varepsilon }{2})\varepsilon 2^{\frac{\left(\varepsilon -1\right)}{2}}}\right]}^{\frac{1}{\varepsilon }},\sigma_{y}=1,\varepsilon =1.5$$

where $Juvenile_{i,j}^{t}$ represents the current location of the *i* the young agents on the *j*th dimension, and $Juvenile_{i,j}^{t+1}$ signifies the new placement of the *i* the young agents on the *j*th dimension. P is the distress coefficient of the young individual, which is a random number within the range of (0,1). $S^{t}$ is the safe position under the current iteration. LF denotes the Lévy flight motion, which is a vector of random numbers based on the Lévy distribution. x and y are two normally distributed vectors, $x∼N(0,\sigma_{x}^{2})$, $y∼N(0,\sigma_{y}^{2})$. $\sigma_{x}$ and $\sigma_{y}$ are the standard deviations.

#### 3.4.2. Foraging Behavior

When the safety signal is relatively low, the herd of walruses chooses to forage. During the foraging process, influenced by risk factors, walrus foraging includes escape and aggregation behaviors.

1. Fleeing behavior

The herd could potentially come under attack from natural enemies when foraging in the deep sea, then they evacuate the area in response to danger signals issued by vigilantes. The escape behavior emerges during the later iterations of WO, and a particular level of perturbation to the herd aids walruses in executing a global quest. Equation (17) simulates the escape behavior of walruses.

(17) $$X_{i,j}^{t+1}=R\times X_{i,j}^{t}-r_{4}^{2}\times \left|X_{best}^{t}-X_{i,j}^{t}\right|$$

where $\left|X_{best}^{t}-X_{i,j}^{t}\right|$ represents the distance between the present individual and the leader agent. $r_{4}$ is a randomly generated number fall within the range of (0,1).

2. Gathering behavior

Gathering behavior occurs when a walrus herd forages in a relatively safe environment. As shown in Equation (18), individuals in the group cooperate in foraging and moving according to the location of other walrus individuals. This group behavior of sharing location information can help walrus herds explore areas with higher food abundance.

(18) $$X_{i,j}^{t+1}=\frac{Position_{i,best}^{t}+Position_{i,\sec ond}^{t}}{2}$$

(19) $$\{\begin{array}{c} Position_{best}^{t}=X_{best}^{t}-a_{1}\times b_{1}\times \left|X_{best}^{t}-X_{i,j}^{t}\right|\\ Position_{\sec ond}^{t}=X_{\sec ond}^{t}-a_{2}\times b_{2}\times \left|X_{\sec ond}^{t}-X_{i,j}^{t}\right| \end{array}$$

(20) $$a=\beta \times (r_{5}-1)$$

(21) b=tan(θ)

where $Position_{i,best}^{t}$ and $Position_{i,\sec ond}^{t}$, respectively, represent the adjusted positions of the current individual influenced by the current optimal ($X_{best}^{t}$) and suboptimal walruses ($X_{\sec ond}^{t}$). a and b are clustering coefficients, calculated by Equations (20) and (21), respectively. $r_{5}$ is a r a random number within (0,1). The value range of θ is (0,π).

## 4. The Proposed Approach

This part introduces a refined binary walrus optimization algorithm BGEPWO, which utilizes population regeneration mechanism, EOBL strategy, and golden sine perturbation. Firstly, the population is initialized using ICMIC chaotic mapping, replacing the random initialization in the original algorithm. Secondly, the BGEPWO algorithm introduces an adaptive operator to obtain a new adaptive safety signal. Thirdly, a population regeneration mechanism has been added to generate promising new individuals while eliminating old and weak ones. Fourthly, the EOBL strategy is adopted in the escape behavior of walruses to guide their escape direction. Fifthly, BGEPWO uses the golden sine strategy to perturb the population at the late stage of each iteration. Finally, BGEPWO, for FS is provided in a binary format.

### 4.1. ICMIC Chaotic Mapping

In heuristic algorithms, the distribution of the population at its initial position can affect the precision and efficiency of global optimization, while in WO algorithms, the initial population is obtained through random initialization. This random initialization reduces the diversity of solutions, making the algorithm prone to stuck in local optimal traps. It has been experimentally proved that population initialization using chaotic mapping can impact the entire algorithmic process, and typically produce superior outcomes compared with random initialization [77].

ICMIC has a higher Lyapunov exponent; therefore, it exhibits more pronounced chaotic features compared with other chaotic maps [78].

In this study, ICMIC chaotic mapping is introduced in the initialization phase to expand the dispersion of the population and enhance the efficacy of the initial solution. The ergodicity of ICMIC chaotic mapping enables better diversity in the beginning phase of the walrus group, avoiding premature convergence and improving the accuracy and convergence of global optimization, overcoming the shortcomings of traditional optimization algorithms.

Equation (22) describes the mathematical expression of ICMIC chaotic mapping.

(22) $$x_{i+1}=ICMIC\left(x_{i}\right)=\sin\left(\frac{\mu }{x_{i}}\right)$$

where $x_{i}\in [-1,0)\cup (0,1]$, μ∈(0,+∞).

### 4.2. Adaptive Safety Signal

The SafetySig in the original WO algorithm is determined by randomly generating a numerical value within (0,1). As SafetySig is an important control signal for selecting the operation process of the algorithm, the complete randomness of the calculation of SafetySig will seriously affect the stability and convergence algorithm’s capability. With the aim of achieving a better convergence trend, it should prioritize exploring the search space during the initial iteration phase and enhancing development capacity in the later iteration phase. Therefore, this paper introduces an adaptive operator to attain a more robust and enhanced equilibrium between exploration and exploitation.

Adaptive operator ω is shown in Equation (24). After adding the adaptive operator, SafetySig is calculated by Equation (23).

(23) $$SafetySig=r_{6}\times \omega$$

(24) $$\omega =\frac{1}{\exp\left(5\times {\left(\frac{t}{T}\right)}^{2}\right)}$$

where t signifies the current iteration count, and T signifies the maximum iteration count.$r_{6}$ is a random number within (0,1).

**Figure 1:**
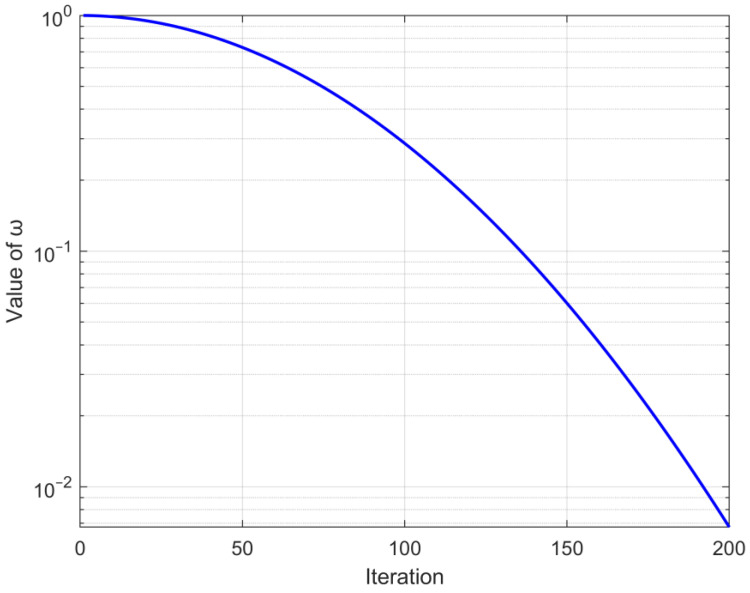
The changing trend of the adaptive operator ω.

**Figure 2:**
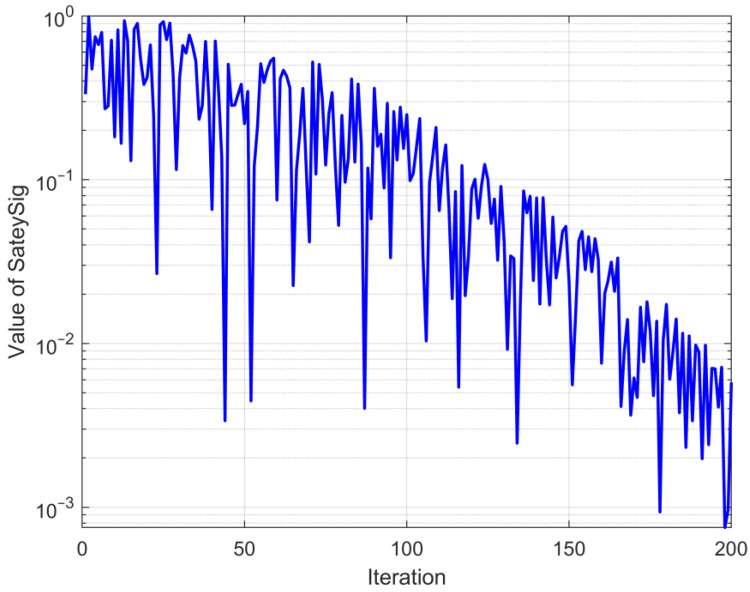
The changing trend of new SafetySig.

The adaptive operator ω converges from 1 to 0 as the iterative process; the trend is shown in Figure 1. The random number $r_{6}$ is used to maintain the randomness of process selection, facilitating the algorithm to avoid local optima. This change in an improved security signal with the iteration is depicted in Figure 2.

It is evident from the trend presented in Figure 2 that the safety signal retains an overall trend of converging from 1 to 0. In the beginning stage of the cycle, it can engage in more habitat behavior, and in the subsequent phase of the iteration, it is able to choose more foraging behavior, which achieves equilibrium between algorithmic exploration and exploitation. Concurrently, the utilization of a random number can play a certain role in perturbation, preventing the algorithm from getting stuck in suboptimal solutions.

### 4.3. Population Regeneration Mechanism

**Figure 3:**
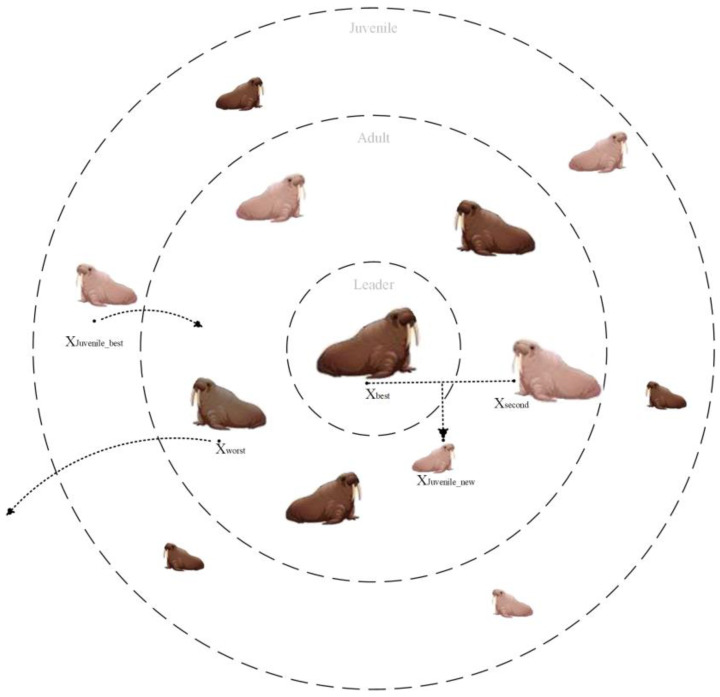
Update of walrus population.

The original WO algorithm simulates the migration, roosting, gathering, or fleeing behavior of a walrus population, ignoring the simulation of the regeneration of the entire population. The old and weak individuals in the walrus herb may be removed from the group due to their own or natural enemies. Meanwhile, new walrus individuals will be generated during the process of roosting in reproduction. In this paper, we simulate the population regeneration mechanism during the roosting process of the walrus population, as shown in Figure 3.

**Figure 4:**
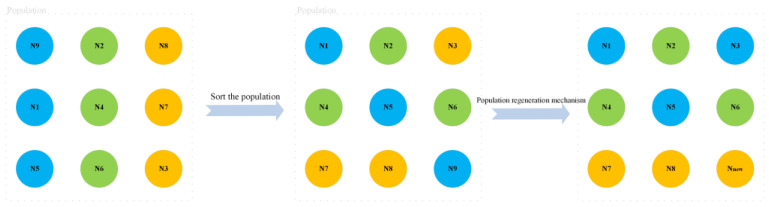
Population regeneration mechanism.

In the regeneration mechanism of the walrus population, the oldest and weakest walruses (i.e., the worst solutions) among the adult walruses (consisting of male and female walruses) are removed, and the strongest (i.e., the optimal solution) juvenile walruses grow up to be the adult walruses. At the same time, a juvenile walrus is bred and added to the population through optimal and suboptimal individuals. The above program is shown in Figure 4.

The simulation of the regeneration mechanism is shown in Equations (25) and (26).

(25) $$X_{worst}^{t}=X_{Juvenile\_best}^{t}$$

(26) $$X_{Juvenile\_best}^{t}=\frac{X_{best}^{t}+X_{\sec ond}^{t}}{2}$$

where $X_{worst}^{t}$ is the worst individual among adult walruses, $X_{Juvenile\_best}^{t}$ is the best individual among juvenile walruses, and $X_{best}^{t}$ and $X_{\sec ond}^{t}$ denote the optimal and suboptimal individuals under the current iteration, respectively.

The roosting process of the original WO algorithm updated the positions of male walruses, female walruses, and juvenile walruses, respectively. The addition of a population regeneration mechanism can enhance the algorithm’s developmental capacity, making the population continuously move toward the optimal solution. Algorithm 1 describes the pseudocode of the population regeneration mechanism.
**Algorithm 1.** Pseudocode of population regeneration mechanism.1: % Initialize the worst walrus position and its fitness value $F_{worst\_mf}$ in adult walruses, and the best walrus position $Index_{best\_j}$ and its fitness value $F_{best\_j}$ in juvenile walruses;2:  Initialize $F_{worst\_mf}$, $Index_{worst\_mf}$, $F_{best\_j}$, $Index_{best\_j}$; 3: % Find the worst individual among adult walruses (consisting of males and females)4:  **For** i from 1 to $n_{male}+n_{female}$
5:    Calculate fitness value $F_{i}$;6:    **If**
$F_{i}>F_{worst\_mf}$ **then**7:      $Index_{worst\_mf}=i$;8:    **End If**9:  **End For**10: % Find the optimal individual among juvenile walruses11: **For** i from $n-n_{Juvenile}+1$ to n
12:   Calculate $F_{i}$;13:**   If**
$F_{i}$ < $F_{best\_j}$
**then**14:     $F_{best\_j}$ = $F_{i}$;15:     $Index_{best\_j}=i$;16:     $X_{Index_{worst\_mf}}=X_{i}$;17:   **End If**18: **End For**19: % Reproduce a juvenile walrus by optimal and suboptimal individuals 20: **If**
$Index_{best\_j}>0$ **then**21:   $X_{Index_{best\_j}}=\frac{X_{best}+X_{\sec ond}}{2}$;22: **End If**

### 4.4. Elite Opposition-Based Learning Strategy

If walruses encounter natural predators while foraging, they leave the immediate area to avoid danger. The escape behavior of the original WO algorithm is simulated as in Equation (17). The position of the walrus during the escape process is influenced by its current place and the place of the leader walrus. However, during the escape process, the location of the leader walrus may not necessarily be the current safest position (i.e., optimal solution).

In this research, EOBL is involved to guide the escape behavior of current walruses by searching for the better position between the leader walrus (i.e., the best solution at present) and the individual in reverse. The process is simulated as shown in the following equations:

(27) $$X_{i,j}^{t+1}=R\times X_{i,j}^{t}-r_{4}^{2}\times \left|{X_{best}^{t}}^{*}-X_{i,j}^{t}\right|$$

(28) $${X_{best}^{t}}^{*}=\{\begin{array}{c} X_{best}^{t},f(X_{best}^{t})>f({\bar{X}}_{best}^{t})\\ {\bar{X}}_{best}^{t},\begin{array}{cc} \begin{array}{cc} & else \end{array} & \end{array} \end{array}$$

(29) $${\bar{X}}_{i,j}^{t}=k\times (ub_{j}+lb_{j})-X_{i,j}^{t}$$

where ${X_{best}^{t}}^{*}$ denotes the current optimal solution after the EOBL strategy obtained by Equation (28). ${\bar{X}}_{best}^{t}$ is the reverse solution of $X_{best}^{t}$, calculated by Equation (29). k is the dynamic coefficient within (0,1). The dynamic bounds are given by $ub_{j}$ and $lb_{j}$.

By introducing the EOBL strategy, it is feasible to flee from the current local optimal trap, expand the scope of exploration, and enhance the variety of the population.

### 4.5. Golden Sine Disturbance

Throughout the iteration process, to prevent the algorithm from getting stuck in local optimal solutions as well as enhance the population’s ability to explore, this research employed the golden sine strategy to disturb the population at the conclusion of each iteration [79]. The disturbance method is shown in Equation (30).

(30) $$X_{i,j}^{t+1}=X_{i,j}^{t}\times \left|\sin(R_{1})\right|+R_{2}\times \sin(R_{1})\times \left|x_{1}\times X_{best}^{t}-x_{2}\times X_{i,j}^{t}\right|$$

where $R_{1}$ represents a randomly generated value within [0,2π], which dictates the distance an individual moves during each iteration. $R_{2}$ is a stochastic value in the range of [0,π] that dictates the direction for updating the current individual in each iteration. $x_{1}$ and $x_{2}$ are coefficients derived from the golden section.

By the golden sine disturbance of individual positions, the algorithm is able to conduct a more comprehensive search of the area during the whole iteration, improve the capacity for exploration, and efficiently address the issue of being caught in local traps in order to increase the algorithm’s convergence rate.

### 4.6. Fitness Function

The primary objective of FS is to identify significant features and decrease the dataset’s dimensionality. The better the effectiveness of optimization algorithms in dealing with FS problems, the higher the accuracy of classification. Equation (31) shows the fitness function used here.

(31) F(X)=1−Acc(X)

where Acc(X) is the accuracy of the feature subset X.

### 4.7. Overall Procedure

**Figure 5:**
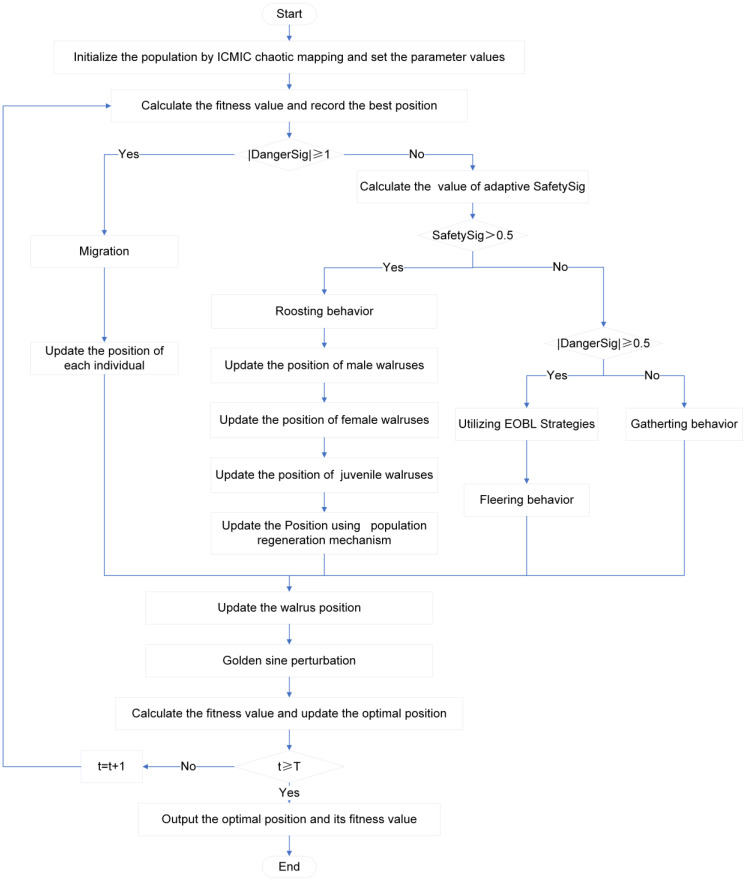
Algorithm flow chart.

The general procedure of the BGEPWO algorithm suggested in this research is depicted Figure 5 and Algorithm 2. Firstly, the population is initialized using ICMIC chaotic mapping, and the relevant parameters are defined. Then, the fitness value is computed, and the current optimal solution is obtained. During the iteration, if |DangerSig|≥1, the walrus herd needs to migrate to a safe area, and the algorithm enters the exploration stage, where the location of each walrus is revised; otherwise, it begins the exploitation stage. If SafetySig≥ 0.5, the walrus herd chooses to roost, at which time the positions of male walruses, female walruses, and juvenile walruses are updated separately, and then a population regeneration mechanism is introduced to eliminate and generate the individual in the group. If SafetySig<0.5, the walrus population starts foraging. During the foraging behavior, if |DangerSig|≥0.5, walruses need to escape the current area. At this time, an EOBL strategy is used to obtain the position of the leader walrus that affects the escape route, and then the walruses’ position is updated. If |DangerSig|<0.5, the walruses continuously move toward the food-intensive area and update their position. At the conclusion of each iteration, the golden sine strategy is utilized to perturb the position of the walruses. Afterwards, the fitness value is calculated, and the current optimal solution is updated. The optimal solution is returned upon the conclusion of the iteration.
**Algorithm 2.** Pseudocode of BGEPWO**Input**: Parameters 1:  Initialize the population by ICMIC chaotic mapping in Algorithm 1;2:  Specify the relevant parameters;3:  Calculate the fitness values, acquire the optimal solution;4:  **While** (t<T) **do**5:   **If** |DangerSig|≥1| **then** {Exploration phase} % Migration6:     Utilize Equation (9) to calculate every individual’s position;7:   **Else** {Exploitation phase} % Reproduction 8:     **If** SafetySig≥ 0.5 **then** % Roosting process 9:       **For** every male individual10:        Update new position based on Halton sequence;11:      **End For**12:      **For** every female individual13:       Update new position by Equation (12);14:**      End For**15:      **For** every juvenile individual16:       Update new position by Equation (13);17:      **End For**18:    Update the population using the mechanism in Algorithm 2;19:    **Else** % Foraging process 20:      **If**
|DangerSig|≥0.5 **then** % Fleeing process 21:       Update new position of leader walrus by Equation (28);22:       Update new position of each walrus by Equation (27);23:      **Else** % Gathering process 24:       Update new position of each walrus using Equation (18);25:      **End If**26:    **End If**27:  **End If**28:  Update the position; 29:  Disturb the population using Golden sine strategy using Equation (30);30:  Compute the fitness value and refine the optimal solution at present; 31:  t = t + 1;32: **End While****Output**: the optimal solution

### 4.8. Binary Mechanism Sigmoid

Metaheuristic algorithms are mostly applied for addressing continuous optimization issues, whereas the solution space of a formulated FS problem is discrete. Thus, a transfer function needs to be inaugurated to convert the solution from continuous domain to discrete domain. In this paper, the Sigmoid function, a commonly used S-shaped transfer function [80], is employed to discretize the continuous algorithm into a binary format BGEPWO, which is capable of addressing feature selection issues using the following equation:

(32) $$S\left(X_{i,j}\right)=\frac{1}{1+e^{-X_{i,j}}}$$

where $X_{i,j}$ and $S\left(X_{i,j}\right)$, respectively, express the position of the ith individual and the probability of changing its binary position.

Then, a threshold r is set. In the present investigation, the threshold r is set to 0.5 to ensure the uniform distribution of 0 and 1 in discrete space. The position is revised with the use of Equation (33).

(33) $$B_{i,j}=\{\begin{array}{c} 0\;\;if\;S\left(X_{i,j}\right)<0.5\\ 1\;\;if\;S\left(X_{i,j}\right)\geq 0.5 \end{array}$$

## 5. Experiments and Discussion

This part first depicts the datasets, evaluation criteria, and experimental configuration utilized during the experiment and conducts experiments and discussions on parameter settings. Then, the BGEPWO is evaluated experimentally against the original BWO algorithm as well as the comparison algorithms. The experimental outcomes are analyzed, and the limitations are pointed out.

### 5.1. Datasets

We selected 21 datasets to evaluate the effectiveness of the BGEPWO algorithm suggested in this study on FS. These datasets were obtained from the UCI machine learning database, OPENML, and Arizona State University (ASU) [81,82,83,84,85]. The details can be found in Table 1, which mainly describes the instances, features, classes, and sources.

### 5.2. Experimental Design

#### 5.2.1. Fitness Function Value

For the purpose of assessing the efficacy of the BGEPWO algorithm suggested in this study for FS, comparative experiments were executed on the BGEPWO algorithm, BWO algorithm, and the binary versions of ten metaheuristic algorithms. Based on the standards of being widely cited, researched, classic algorithms, and recently proposed [86], we selected 10 metaheuristic algorithms and used their binary versions as comparison algorithms. They are BABC, BPSO, BBA, BWOA, BKOA, BSSA, BNOA, BHHO, BCPO, BCOA.

The evaluation indicators for comparative experiments are Fitness function value, Feature subset size, and *F*1-*score*. In addition, the 5% Wilcoxon rank-sum test is involved to verify statistical significance in this paper. The assessment metrics are presented as follows:

1. Fitness function value

As shown in Equation (31), Acc(X) signifies the accuracy of the feature subset X, calculated by the following equation:

(34) $$Acc=\frac{TP+TN}{TP+TN+FP+FN}$$

where TP, TN, FP, and FN represent true positives, true negatives, false positives, and false negatives, respectively.

2. Number of selected features

It denotes the quantity of features selected during the FS process.

3. *F*1-*score*

It is a blend of precision and recall that is utilized for evaluating the quality of predictive models. The formulae of precision and recall are as follows:

(35) $$\mathrm{Pr}ecision=\frac{TP}{TP+FP}$$

(36) Recall=TPTP+FN

*F*1-*score* is the harmonic mean of recall and precision. For binary classification problems, *F*1-*score* is determined using the formula below:

(37) F1=2×Precision×RecallPrecision+Recall

In multiclass classification problems, *Macro*_*F*1-*score* is used as an evaluation indicator [19]. For an N-classification problem, the calculation formula is

(38) $$F1=\frac{1}{N}\sum_{i=1}^{N}F1_{i}$$

where $F1_{i}$ represents the *F*1-*score* for the *i*th category.

In addition, the 5% Wilcoxon rank-sum test is involved to verify whether there is a significant difference between BGEPWO and competitive algorithms for feature selection on the dataset.

#### 5.2.2. Experimental Configuration

The experiments were conducted using identical computers and environmental settings, which use Intel Core i7, 2.8 GHz CPU, and 16 GB of RAM. All experiments were implemented in MATLAB 2023a and run on the same computer with the Windows 10 operating system. KNN (k = 10) was used as the classifier [87].

In this study, we performed two sets of trials. The first set of trials selects the most suitable value of the parameter for processing FS of the BWO and BGEPWO. The second set of trials is the contrast of the effectiveness of BGEPWO with BWO and the comparison algorithms on the dataset.

### 5.3. Parameter Settings

**Figure 6:**
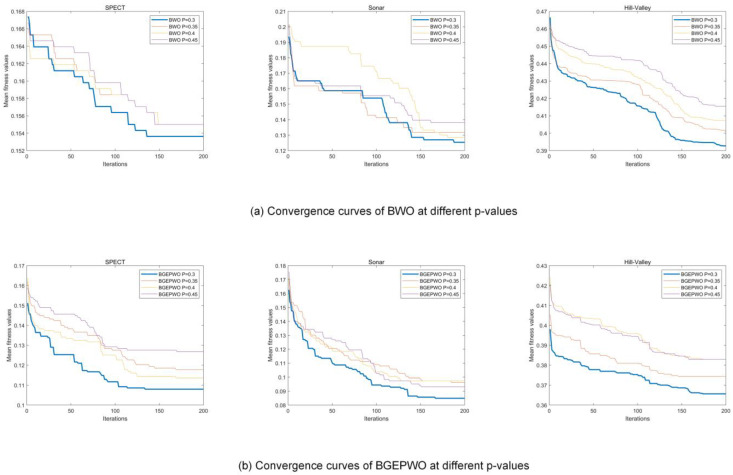
Convergence curves of BWO and BGEPWO at different p values.

We conducted trials on three distinct datasets with different dimensions, Sonar, SPECT, and Hill Valley, to select the optimal parameter settings for BGEPWO and BWO algorithms during FS. We examined four values for the proportion p of male walruses in a herd: $\left\{0.3,0.35,0.4,0.45\right\}$ setting the maximum number of iterations to 200, the population size to 20, and the number of independent runs to 20. The experiment was analyzed based on the mean fitness values and convergence curves. Table 2 presents the mean fitness values of BWO and BGEPWO when using four different values on three datasets. Figure 6a,b displays the curves of convergence of the BWO algorithm and BGEPWO algorithm when using four different values on the aforementioned datasets, respectively. The findings from the experiment indicate that in the case where BWO and BGEPWO have the best results, p is recommended to be set to [0.3].

In the empirical assessment of each algorithm, the relevant parameters were set as depicted in Table 3. The parameter values were set based on their original paper. To ensure fairness, the common parameter settings for all algorithms conducted on the dataset are the same, with 30 individuals in the population, a maximum of 200 iterations, and 20 independent runs.

### 5.4. Experimental Results and Discussions

In this section, the performance of BGEPWO was evaluated and compared with the original BWO and ten other representative metaheuristic algorithms, shown in Table 4, Table 5, Table 6 and Table 7. The table shows the mean, standard deviation, best value, and ranking for different algorithms for 20 independent runs on the datasets in Table 4 and Table 5. The ranking is based on the mean of the evaluation criteria, and the best rankings are highlighted in bold.

Table 4 shows the performance of BGEPWO and competitive algorithms on the fitness function. Overall, BGEPWO achieved the best average fitness value on 18 out of 21 datasets, ranking second on the Arrhythmia and Micro Mass datasets, and eighth on the Zoo dataset, indicating that BGEPWO exhibits strong FS ability on the dataset. The standard deviation and optimal values show that BGEPWO performs relatively consistently and is capable of attaining the highest possible optimal across the majority of the datasets. The mean fitness value of BGEPWO ranked 1.4 on all datasets, which is the highest among all the evaluated algorithms, indicating that the BGEPWO algorithm has a strong capability of searching solution space.

In addition, the BGEPWO algorithm achieved better fitness values compared with the original BWO on all datasets, indicating that our improvements of the algorithm for FS applications are effective.

**Figure 7:**
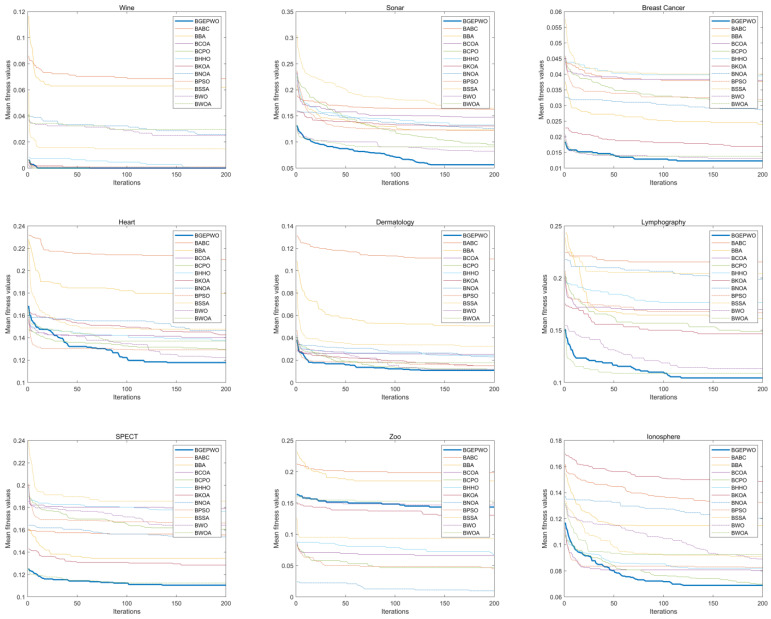
Convergence curves of different algorithms on low-dimensional datasets.

**Figure 8:**
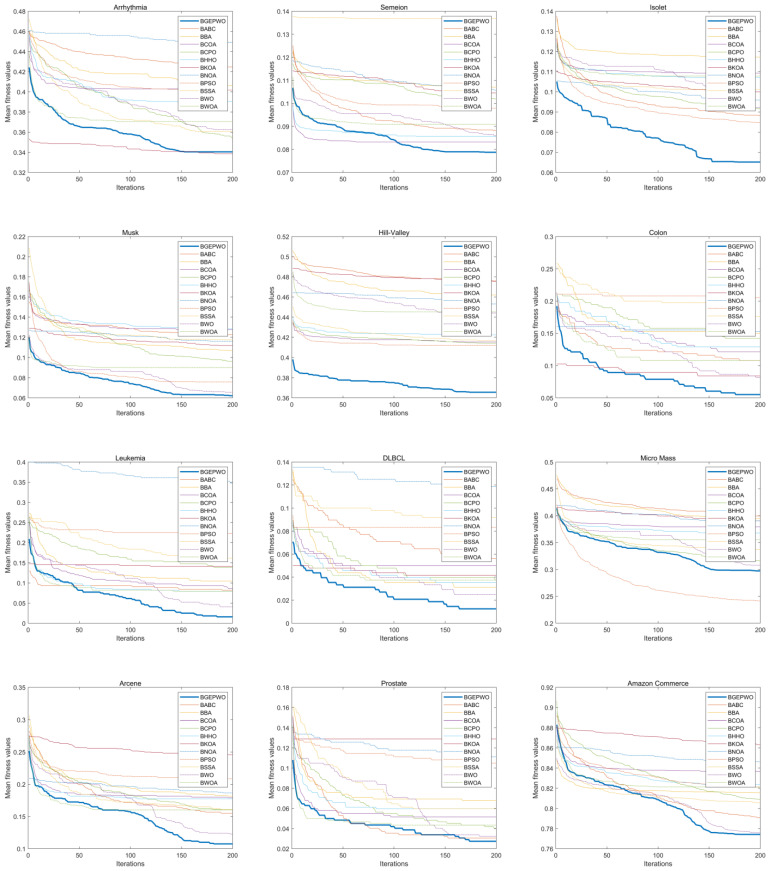
Convergence curves of different algorithms on high-dimensional datasets.

Figure 7 and Figure 8 show the convergence of these algorithms based on the fitness function on low- and high-dimensional datasets, respectively. The convergence curve exhibits that the BGEPWO algorithm demonstrates superior convergence capability among the competing algorithms on 21 datasets. On the Wine, Dermatology, and SPECT datasets, the proposed algorithm converged quickly toward the optimal solution in the preiteration period. On the Sonar, Isolet, Musk, Hill-Valley, and DLBCL datasets, the BGEPWO algorithm not only quickly converged to the optimal solution in the preiteration period but also showed strong convergence ability in the late iteration period. The BGEPWO algorithm did not have the best convergence ability in the early stages on the Breast Cancer, Heart, Lymphography, Semeion, Ionosphere, Amazon, Arcene, Leukemia, Prostate, and Colon datasets, but with its later convergence ability, it demonstrated superior performance compared with other algorithms at the conclusion of the iteration. The proposed algorithm performed only slightly worse than BKOA on the Arrhythmia dataset, but its ability to continuously approach global optima in the later stages could still be observed. The BGEPWO algorithm had also demonstrated excellent convergence ability on the Micro Mass dataset, but its performance was somewhat inferior to that of BPSO. On the Zoo dataset, the proposed BGEPWO performed poorly in both the early and late stages of iteration.

**Figure 9:**
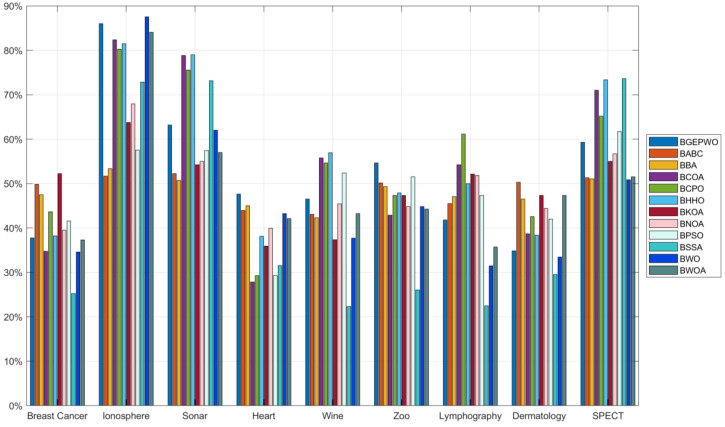
Feature reduction rate on low-dimensional datasets.

**Figure 10:**
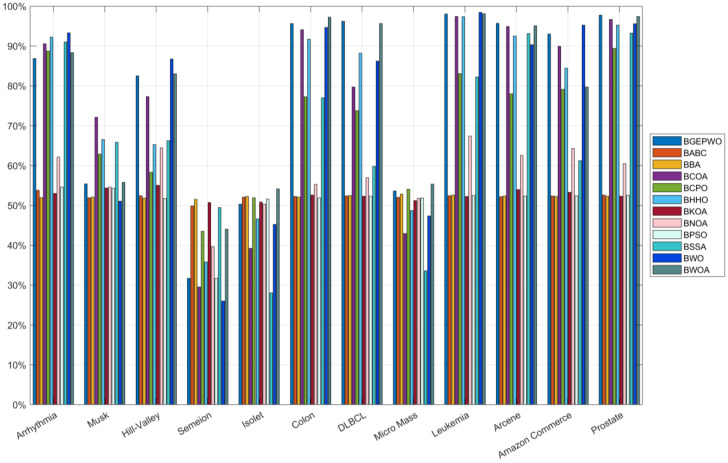
Feature reduction rate on high-dimensional datasets.

Table 5 shows the average number, the average feature reduction rate, and the least number of features selected by the experimental algorithm during the FS process. The mean number of features chosen by BGEPWO on 21 datasets ranked first among all algorithms. There are certain contradictions and trade-offs between accuracy and the feature subset size in the FS process. In a general evaluation system, accuracy is the first consideration, and the quantity of features that have been chosen should be minimized on the basis of ensuring accuracy. The fitness function formula indicates that the lower the value, the higher the accuracy. Based on Table 4 and Table 5 comprehensively, the algorithms whose combined ranking of the mean number of selected features was not worse than BGEPWO had a much worse average fitness value compared with BGEPWO, which means that the algorithms that chose the least number of features in the experiments achieved a poorer accuracy. Figure 9 and Figure 10 show the feature reduction rates obtained by all algorithms on low- and high-dimensional datasets, respectively. The BGEPWO algorithm had the best performance among all the algorithms with an average feature approximation rate of 67.07% on all the datasets, although it only selected the minimum number of features on 5 datasets. Comprehensive analysis shows that on these 21 datasets, the BGEPWO algorithm selected a relatively small size of feature subset on the basis of ensuring a relatively best classification ability, which proves that BGEPWO has a strong feature approximation ability.

Table 6 shows the *F*1-*score* procured in the FS process. BGEPWO achieved optimal performance on 16 datasets, marginally below BABC on the Amazon dataset, lower than the BCPO and BABC algorithms on the Isolet dataset, and lower than the BCPO and BPSO algorithms on the Musk dataset. The proposed algorithm achieved general results on the Zoo and Lymphography datasets, ranking sixth and seventh, respectively. In addition, the BGEPWO algorithm achieved higher performance than BWO on all datasets except the Zoo dataset, indicating that the model using the improved BGEPWO algorithm is more robust. Based on its performance on all datasets, BGEPWO had the best average ranking among all algorithms, signifying that BGEPWO had a better performance and could obtain better predictive classification models.

In order to measure the difference between the proposed BGEPWO and the competitive algorithms from a statistical perspective, we applied a 5% Wilcoxon rank-sum test. Table 7 displays the outcome of comparing the competitive algorithms with BGEPWO, and the symbols +, =, − are used to indicate that the proposed BGEPWO performs “significantly better than, similarly to, or worse than” the compared algorithms, respectively. It can be seen from Table 7 that BGEPWO outperformed the comparison algorithms in the majority of cases. The BGEPWO significantly outperformed the comparison algorithm on 19, 20, 18, 16, 19, 18, 20, 17, 19, 17, 12 datasets, respectively, and the median performance was similar to that of the comparison algorithm on 2, 1, 2, 4, 1, 2, 0, 2, 1, 4, 9 datasets. The BGEPWO algorithm was inferior to the BPSO algorithm only on the Micro Mass dataset, whereas the proposed algorithm performed average on the Zoo dataset, and median performance was worse than the BCOA, BCPO, BHHO, BKOA, BNOA, and BPSO algorithms.

The evaluation results on the datasets indicate that BGEPWO is significantly superior to the original algorithm and ten representative competitive algorithms in terms of fitness function, number of selected features, and *F*1-*score*. The proposed algorithm can fully search the solution space and find the optimal solution, thus achieving excellent performance in fitness function and selected features. The application of multiple strategies can effectively improve the balance between exploration and exploitation, and the involvement of the population regeneration mechanism can enhance convergence ability. Therefore, the BGEPWO algorithm has demonstrated strong convergence ability in experiments. The use of adaptive operators increases the stability of the algorithm, so the proposed algorithm can achieve better results on *F*1-*score*. In addition, 5% of the Wilcoxon rank-sum test findings indicate that BGEPWO is statistically significantly better than the comparison algorithms. The experimental results confirm that BGEPWO has excellent performance and feature reduction ability, as well as strong convergence ability in feature selection applications.

Comparative experiments with the latest metaheuristic algorithms, such as KOA, NOA, CPO, and COA, showed that the original WO algorithm has certain superiority in handling feature selection tasks, and the proposed BGEPWO algorithm outperforms these algorithms significantly on all evaluation indicators. In addition, compared with the results of recent and popular research on the same datasets dealing with feature selection problems, the BGEPWO algorithm achieved higher accuracy and performance than the MPPSO, HGSA (using KNN), and SDBA algorithms on 80%, 67%, and 80% of datasets, respectively, indicating that the proposed algorithm has certain competitiveness and advantages in feature selection applications.

### 5.5. Limitations of BGEPWO

Despite the experimental simulation results demonstrating the superiority of the suggested BGEPWO over some comparative algorithms in dealing with FS problems, there are still some limitations.

Compared with competitive algorithms, BGEPWO struggled to identify the smallest set of features in the majority of datasets. Although it is not the most important indicator for the algorithm in FS, and a blind reduction in the feature subset size may seriously affect classification accuracy, it is still an important indicator that we need to refer to when dealing with FS problems. Therefore, a novel strategy may be employed to improve BGEPWO to select fewer features while ensuring classification accuracy.

In addition, the algorithm may not always converge the fastest in early iterations, while it exhibits robust convergence ability in later iterations. This indicates that the algorithm requires sufficient iterations to achieve impressive results. Consequently, the algorithm is well suited for analyzing datasets with sufficient computational iteration time and is not conducive to environments necessitating expeditious results.

## 6. Conclusions

This study proposes the BGEPWO algorithm based on the WO algorithm to tackle FS issues. The BGEPWO algorithm uses the following five new strategies: initializing the population using ICMIC chaotic mapping, introducing an adaptive operator to improve safety signals, proposing a population regeneration mechanism to eliminate old and weak individuals and generate new promising ones, using the EOBL strategy to guide the walruses’ escape behavior, and applying the golden sine strategy to perturb the population. The proposed BGEPWO algorithm was validated and evaluated on 21 datasets, compared with the original BWO algorithm and 10 representative competitive algorithms in terms of fitness value and number of selected features, and a 5% Wilcoxon rank-sum test was used for validation. The results indicate that BGEPWO significantly enhanced the original algorithm’s ability to deal with FS issues. Moreover, in most cases, the BGEPWO algorithm significantly outperformed the other 11 algorithms on these evaluation criteria.

However, the BGEPWO algorithm still needs further research and improvement to minimize the quantity of chosen features while maintaining precision. In the future, we intend to utilize the algorithm for addressing various real-world issues, including image processing and fault diagnosis. The effectiveness of the BGEPWO algorithm can also be examined using various classification methods. In addition, we plan to explore novel metaheuristic algorithms for integration in order to address a broader spectrum of issues.

## Figures and Tables

**Table 1:** Datasets information.

Datasets	Instances	Features	Classes	Sources
Breast Cancer	569	30	2	UCI
Ionosphere	351	34	2	UCI
Sonar	208	60	2	UCI
Heart	270	13	2	UCI
Wine	178	13	3	UCI
Zoo	101	17	7	UCI
Lymphography	148	18	4	UCI
Arrhythmia	452	279	16	UCI
Musk 1	476	166	2	UCI
Hill-Valley	606	100	2	UCI
Semeion Handwritten Digit	1953	256	10	UCI
Dermatology	366	34	6	UCI
SPECT	267	22	2	UCI
Isolet	1560	617	26	ASU
Colon	62	2000	2	OPENML
DLBCL	77	5469	2	OPENML
Micro Mass	571	1300	20	OPENML
Leukemia	72	7129	2	OPENML
Arcene Cancer Dataset	200	10,000	2	OPENML
Amazon	1500	10,000	50	OPENML
Prostate Tumors	102	12,600	2	OPENML

**Table 2:** The mean fitness values of different p values.

Data Set	Method	*p =* 0.3	*p =* 0.35	*p =* 0.4	*p =* 0.45
Sonar	BWO	0.1254	0.1317	0.1286	0.1381
	BGEPWO	0.0849	0.0963	0.0974	0.0931
SPECT	BWO	0.1536	0.1550	0.1550	0.1550
	BGEPWO	0.1080	0.1177	0.1136	0.1267
Hill-Valley	BWO	0.3927	0.4008	0.4073	0.4155
	BGEPWO	0.3657	0.3745	0.3830	0.3830

**Table 3:** Experimental parameter settings.

Algorithm	Parameter	Value
BPSO	ω	[0.2, 0.9]
	$c_{1}$	2
	$c_{1}$	2
BABC	ratio	0.6
BSSA	Leader position update probability	0.5
BWOA	Convergence constant	[2, 0]
	Spiral factor	1
BBA	$Q_{\min}$	0
	$Q_{\max}$	2
BCPO	T	2
	α	0.1
	$T_{f}$	0.5
BHHO	$E_{0}$	(−1, 1)
	$E_{1}$	[2, 0]
BNOA	δ	0.05
	$Pa_{1}$	0.2
	$Pa_{1}$	0.4
KOA	Constant	15
	Initial gravitational value	0.1
	Control parameter	3
BWO	*p*	0.3
BGEPWO	*p*	0.3

**Table 4:** Comparison results in terms of fitness values between BGEPWO and competitive algorithms.

		BABC	BBA	BCOA	BCPO	BHHO	BKOA	BNOA	BPSO	BSSA	BWOA	BWO	BGEPWO
Wine	Mean	0.0685	0.0620	0.0000	0.0000	0.0000	0.0000	0.0259	0.0009	0.0148	0.0296	0.0250	0.0000
	Std	0.0544	0.0227	0.0000	0.0000	0.0000	0.0000	0.0111	0.0041	0.0076	0.0093	0.0091	0.0000
	Best	0.0185	0.0185	0.0000	0.0000	0.0000	0.0000	0.0185	0.0000	0.0000	0.0185	0.0185	0.0000
	Rank	12	11	**1**	**1**	**1**	**1**	9	6	7	10	8	**1**
Sonar	Mean	0.1627	0.1230	0.1476	0.0944	0.1325	0.1310	0.1262	0.1222	0.1651	0.0905	0.0817	0.0563
	Std	0.0589	0.0291	0.0237	0.0159	0.0208	0.0162	0.0265	0.0318	0.0529	0.0207	0.0254	0.0215
	Best	0.0317	0.0794	0.0794	0.0635	0.0952	0.0952	0.0794	0.0476	0.0952	0.0476	0.0317	0.0159
	Rank	11	6	10	4	9	8	7	5	12	3	2	**1**
Breast Cancer	Mean	0.0377	0.0240	0.0380	0.0313	0.0395	0.0170	0.0287	0.0319	0.0401	0.0137	0.0132	0.0123
	Std	0.0207	0.0080	0.0030	0.0029	0.0037	0.0042	0.0037	0.0030	0.0069	0.0034	0.0032	0.0026
	Best	0.0058	0.0117	0.0351	0.0292	0.0351	0.0117	0.0234	0.0292	0.0292	0.0058	0.0058	0.0058
	Rank	9	5	10	7	11	4	6	8	12	3	2	**1**
Heart	Mean	0.2099	0.1796	0.1401	0.1296	0.1377	0.1426	0.1463	0.1290	0.1475	0.1370	0.1222	0.1179
	Std	0.0633	0.0440	0.0092	0.0063	0.0100	0.0094	0.0176	0.0063	0.0225	0.0179	0.0233	0.0198
	Best	0.0988	0.1358	0.1235	0.1235	0.1235	0.1235	0.1111	0.1235	0.1235	0.0988	0.0988	0.0988
	Rank	12	11	7	4	6	8	9	3	10	5	2	**1**
Dermatology	Mean	0.1105	0.0505	0.0250	0.0114	0.0241	0.0150	0.0227	0.0150	0.0323	0.0177	0.0118	0.0109
	Std	0.0765	0.0205	0.0050	0.0040	0.0080	0.0085	0.0086	0.0068	0.0108	0.0081	0.0060	0.0037
	Best	0.0091	0.0182	0.0182	0.0091	0.0091	0.0000	0.0091	0.0000	0.0091	0.0091	0.0091	0.0091
	Rank	12	11	9	2	8	4	7	4	10	6	3	**1**
Arrhythmia	Mean	0.4246	0.4063	0.4018	0.3537	0.3904	0.3386	0.4493	0.4018	0.3607	0.3706	0.3625	0.3404
	Std	0.0476	0.0163	0.0197	0.0201	0.0247	0.0091	0.0098	0.0196	0.0406	0.0152	0.0217	0.0129
	Best	0.3456	0.3750	0.3603	0.3088	0.3309	0.3088	0.4338	0.3603	0.3162	0.3456	0.3088	0.3162
	Rank	11	10	8	3	7	**1**	12	8	4	6	5	2
Lymphography	Mean	0.2156	0.1611	0.1700	0.1489	0.1767	0.1467	0.1989	0.1667	0.2044	0.1089	0.1133	0.1044
	Std	0.0645	0.0259	0.0220	0.0146	0.0210	0.0254	0.0210	0.0153	0.0266	0.0142	0.0189	0.0163
	Best	0.0889	0.1111	0.1333	0.1333	0.1333	0.0889	0.1556	0.1333	0.1333	0.0889	0.0667	0.0889
	Rank	12	6	8	5	9	4	10	7	11	2	3	**1**
SPECT	Mean	0.1556	0.1346	0.1790	0.1605	0.1765	0.1284	0.1537	0.1660	0.1858	0.1123	0.1642	0.1105
	Std	0.0387	0.0160	0.0075	0.0113	0.0107	0.0109	0.0102	0.0102	0.0141	0.0089	0.0139	0.0075
	Best	0.0988	0.0988	0.1605	0.1358	0.1481	0.1111	0.1358	0.1481	0.1605	0.0988	0.1481	0.0988
	Rank	6	4	11	7	10	3	5	9	12	2	8	**1**
Zoo	Mean	0.1984	0.1855	0.0661	0.0468	0.0677	0.1306	0.0097	0.0452	0.0935	0.1532	0.1468	0.1435
	Std	0.0471	0.0231	0.0127	0.0165	0.0144	0.0195	0.0152	0.0193	0.0099	0.0143	0.0195	0.0245
	Best	0.1290	0.1613	0.0323	0.0323	0.0323	0.0968	0.0000	0.0323	0.0645	0.1290	0.0968	0.0968
	Rank	12	11	4	3	5	7	**1**	2	6	10	9	8
Semeion	Mean	0.0882	0.1070	0.0833	0.1020	0.0857	0.1045	0.1058	0.0980	0.1369	0.0909	0.0858	0.0788
	Std	0.0073	0.0097	0.0042	0.0040	0.0053	0.0046	0.0053	0.0293	0.0214	0.0043	0.0090	0.0077
	Best	0.0774	0.0900	0.0753	0.0962	0.0774	0.0983	0.0962	0.0774	0.0962	0.0837	0.0669	0.0649
	Rank	5	11	2	8	3	9	10	7	12	6	4	**1**
Isolet	Mean	0.0884	0.1172	0.1093	0.0916	0.1071	0.1000	0.0959	0.0848	0.1012	0.1079	0.0921	0.0652
	Std	0.0053	0.0096	0.0038	0.0047	0.0054	0.0041	0.0051	0.0067	0.0080	0.0057	0.0120	0.0101
	Best	0.0791	0.0983	0.1026	0.0833	0.0962	0.0897	0.0897	0.0748	0.0791	0.0983	0.0662	0.0534
	Rank	3	12	11	4	9	7	6	2	8	10	5	**1**
Ionosphere	Mean	0.1325	0.1146	0.0802	0.0698	0.0821	0.1486	0.1203	0.0825	0.0910	0.0925	0.0887	0.0689
	Std	0.0491	0.0116	0.0108	0.0077	0.0102	0.0133	0.0237	0.0060	0.0168	0.0125	0.0148	0.0087
	Best	0.0566	0.0849	0.0566	0.0566	0.0566	0.1226	0.0755	0.0755	0.0660	0.0755	0.0566	0.0566
	Rank	11	9	3	2	4	12	10	5	7	8	6	**1**
Musk	Mean	0.1227	0.1066	0.1276	0.0965	0.1283	0.1119	0.1161	0.0759	0.1175	0.0902	0.0657	0.0622
	Std	0.0510	0.0118	0.0214	0.0145	0.0149	0.0109	0.0048	0.0194	0.0293	0.0106	0.0167	0.0145
	Best	0.0629	0.0909	0.0909	0.0629	0.0979	0.0979	0.1119	0.0350	0.0839	0.0699	0.0350	0.0280
	Rank	10	6	11	5	12	7	8	3	9	4	2	**1**
Hill-Valley	Mean	0.4761	0.4621	0.4154	0.4140	0.4225	0.4755	0.4530	0.4121	0.4168	0.4453	0.4442	0.3657
	Std	0.0380	0.0133	0.0088	0.0057	0.0094	0.0079	0.0111	0.0056	0.0167	0.0079	0.0101	0.0079
	Best	0.4286	0.4451	0.4011	0.4011	0.4011	0.4615	0.4286	0.4011	0.3846	0.4286	0.4286	0.3516
	Rank	12	10	4	3	6	11	9	2	5	8	7	**1**
Amazon	Mean	0.7908	0.8157	0.8368	0.8090	0.8236	0.8632	0.8462	0.8211	0.8057	0.8213	0.7756	0.7742
	Std	0.0163	0.0164	0.0147	0.0124	0.0143	0.0074	0.0098	0.0140	0.0300	0.0184	0.0303	0.0225
	Best	0.7644	0.7578	0.8111	0.7778	0.7956	0.8467	0.8178	0.7933	0.7244	0.7822	0.7044	0.7333
	Rank	3	6	10	5	9	12	11	7	4	8	2	**1**
Arcene	Mean	0.1550	0.1817	0.1792	0.1600	0.1775	0.2458	0.1867	0.2083	0.1608	0.1600	0.1225	0.1075
	Std	0.0144	0.0275	0.0366	0.0250	0.0255	0.0152	0.0103	0.0268	0.0380	0.0356	0.0282	0.0357
	Best	0.1333	0.1167	0.1000	0.1000	0.1333	0.2167	0.1667	0.1667	0.0833	0.0667	0.0667	0.0333
	Rank	3	9	8	4	7	12	10	11	6	4	2	**1**
DLBCL	Mean	0.0563	0.0313	0.0500	0.0375	0.0354	0.0417	0.1188	0.0833	0.0917	0.0375	0.0250	0.0125
	Std	0.0609	0.0379	0.0256	0.0128	0.0280	0.0000	0.0153	0.0000	0.0396	0.0267	0.0209	0.0196
	Best	0.0000	0.0000	0.0000	0.0000	0.0000	0.0417	0.0833	0.0833	0.0000	0.0000	0.0000	0.0000
	Rank	9	3	8	5	4	7	12	10	11	5	2	**1**
Micro Mass	Mean	0.3997	0.3948	0.3794	0.3151	0.3677	0.3907	0.3904	0.2416	0.3375	0.3555	0.3073	0.2974
	Std	0.0855	0.0250	0.0133	0.0144	0.0202	0.0156	0.0089	0.0195	0.0329	0.0194	0.0310	0.0300
	Best	0.2907	0.3314	0.3547	0.2907	0.3314	0.3547	0.3663	0.2151	0.2674	0.3256	0.2500	0.2326
	Rank	12	11	8	4	7	10	9	**1**	5	6	3	2
Leukemia	Mean	0.0818	0.1045	0.0864	0.1386	0.0795	0.1409	0.3477	0.2227	0.1614	0.0795	0.0409	0.0159
	Std	0.0775	0.0573	0.0530	0.0429	0.0414	0.0140	0.0517	0.0203	0.0950	0.0439	0.0358	0.0222
	Best	0.0000	0.0000	0.0000	0.0909	0.0000	0.1364	0.1818	0.1818	0.0455	0.0000	0.0000	0.0000
	Rank	5	7	6	8	3	9	12	11	10	3	2	**1**
Prostate	Mean	0.0306	0.0677	0.0516	0.0403	0.0597	0.1290	0.1113	0.1048	0.0597	0.0435	0.0323	0.0274
	Std	0.0127	0.0275	0.0162	0.0143	0.0216	0.0000	0.0195	0.0206	0.0335	0.0189	0.0105	0.0216
	Best	0.0000	0.0323	0.0323	0.0323	0.0323	0.1290	0.0645	0.0645	0.0323	0.0000	0.0000	0.0000
	Rank	2	9	6	4	7	12	11	10	7	5	3	**1**
Colon	Mean	0.1079	0.1500	0.1211	0.1421	0.1289	0.0842	0.1526	0.2053	0.1763	0.1079	0.0816	0.0553
	Std	0.1189	0.0428	0.0386	0.0386	0.0435	0.0265	0.0162	0.0162	0.0622	0.0435	0.0318	0.0361
	Best	0.0000	0.0526	0.0526	0.0526	0.0526	0.0526	0.1053	0.1579	0.0526	0.0000	0.0000	0.0000
	Rank	4	9	6	8	7	3	10	12	11	4	2	**1**
Average rank		8.4	8.4	7.2	4.6	6.9	7.2	8.8	6.3	8.5	5.6	3.9	**1.4**

**Table 5:** Comparison results in terms of number of selected features between BGEPWO and competitive algorithms.

		BABC	BBA	BCOA	BCPO	BHHO	BKOA	BNOA	BPSO	BSSA	BWOA	BWO	BGEPWO
Wine	Mean	7.40	7.50	5.75	5.90	5.60	8.14	7.10	6.19	10.10	8.10	7.38	6.95
	FRR	43.08%	42.31%	55.77%	54.62%	56.92%	37.36%	45.42%	52.38%	22.34%	37.73%	43.22%	46.52%
	Best	4	5	2	2	3	6	4	4	5	7	4	4
	Rank	8	9	2	3	**1**	11	6	4	12	10	7	5
Sonar	Mean	28.67	29.57	12.71	14.67	12.62	27.48	27.00	25.52	16.10	22.81	25.81	22.10
	FRR	52.22%	50.71%	78.81%	75.56%	78.97%	54.21%	55.00%	57.46%	73.17%	61.98%	56.98%	63.17%
	Best	7	7	5	7	4	7	7	7	5	7	7	7
	Rank	11	12	2	3	**1**	10	9	7	4	6	8	5
Breast Cancer	Mean	15.05	15.76	19.57	16.90	18.52	14.33	18.14	17.52	22.43	19.62	18.81	18.67
	FRR	49.84%	47.46%	34.76%	43.65%	38.25%	52.22%	39.52%	41.59%	25.24%	34.60%	37.30%	37.78%
	Best	7	7	7	7	7	7	7	7	7	7	7	6
	Rank	2	3	10	4	7	**1**	6	5	12	11	9	8
Heart	Mean	7.29	7.14	9.38	9.19	8.05	8.33	7.81	9.19	8.90	7.38	7.52	6.81
	FRR	43.96%	45.05%	27.84%	29.30%	38.10%	35.90%	39.93%	29.30%	31.50%	43.22%	42.12%	47.62%
	Best	5	4	7	5	3	6	4	7	3	3	6	4
	Rank	3	2	12	10	7	8	6	10	9	4	5	**1**
Dermatology	Mean	16.90	18.19	20.86	19.52	20.95	17.90	18.90	19.71	23.95	22.62	17.90	22.14
	FRR	50.28%	46.50%	38.66%	42.58%	38.38%	47.34%	44.40%	42.02%	29.55%	33.47%	47.34%	34.87%
	Best	7	7	7	7	7	7	7	7	7	7	7	7
	Rank	**1**	4	8	6	9	2	5	7	12	11	2	10
Arrhythmia	Mean	128.86	134.00	26.38	31.43	21.71	131.14	105.71	126.76	25.14	18.76	32.52	36.62
	FRR	53.81%	51.97%	90.54%	88.74%	92.22%	53.00%	62.11%	54.57%	90.99%	93.28%	88.34%	86.87%
	Best	7	7	7	7	5	7	7	7	6	5	3	7
	Rank	10	12	4	5	2	11	8	9	3	**1**	6	7
Lymphography	Mean	9.81	9.52	8.24	7.00	9.00	8.62	8.67	9.48	13.95	12.33	11.57	10.48
	FRR	45.50%	47.09%	54.23%	61.11%	50.00%	52.12%	51.85%	47.35%	22.49%	31.48%	35.71%	41.80%
	Best	6	6	2	2	4	6	2	4	3	5	7	5
	Rank	8	7	2	**1**	5	3	4	6	12	11	10	9
SPECT	Mean	10.71	10.76	6.38	7.67	5.86	9.90	9.52	8.43	5.81	10.81	10.67	8.95
	FRR	51.30%	51.08%	71.00%	65.15%	73.38%	54.98%	56.71%	61.69%	73.59%	50.87%	51.52%	59.31%
	Best	7	5	1	5	1	7	6	6	1	5	7	5
	Rank	10	11	3	4	2	8	7	5	**1**	12	9	6
Zoo	Mean	8.48	8.62	9.71	8.95	8.86	8.95	9.38	8.24	12.57	9.38	9.48	7.71
	FRR	50.14%	49.30%	42.86%	47.34%	47.90%	47.34%	44.82%	51.54%	26.05%	44.82%	44.26%	54.62%
	Best	4	7	4	5	6	6	6	5	6	7	7	5
	Rank	3	4	11	6	5	6	8	2	12	8	10	**1**
Semeion	Mean	128.43	124.10	180.33	144.57	164.29	126.14	154.52	174.86	129.43	189.38	143.33	174.86
	FRR	49.83%	51.53%	29.56%	43.53%	35.83%	50.73%	39.64%	31.70%	49.44%	26.02%	44.01%	31.70%
	Best	7	7	7	7	7	7	7	7	7	7	7	7
	Rank	3	**1**	11	6	8	2	7	9	4	12	5	9
Isolet	Mean	295.57	294.48	374.81	296.71	329.33	303.43	306.38	298.67	443.95	338.00	282.90	306.38
	FRR	52.10%	52.27%	39.25%	51.91%	46.62%	50.82%	50.34%	51.59%	28.05%	45.22%	54.15%	50.34%
	Best	7	7	7	7	7	7	7	7	7	7	7	7
	Rank	3	2	11	4	9	6	7	5	12	10	**1**	7
Ionosphere	Mean	16.43	15.86	6.00	6.71	6.29	12.33	10.90	14.43	9.24	4.24	5.43	4.76
	FRR	51.68%	53.36%	82.35%	80.25%	81.51%	63.73%	67.93%	57.56%	72.83%	87.54%	84.03%	85.99%
	Best	7	7	2	3	2	7	2	7	2	1	2	3
	Rank	12	11	4	6	5	9	8	10	7	**1**	3	2
Musk	Mean	79.81	79.57	46.29	61.62	55.52	75.71	75.38	75.86	56.67	81.24	73.33	74.05
	FRR	51.92%	52.07%	72.12%	62.88%	66.55%	54.39%	54.59%	54.30%	65.86%	51.06%	55.82%	55.39%
	Best	7	7	7	7	7	7	7	7	7	7	7	7
	Rank	11	10	**1**	4	2	8	7	9	3	12	5	6
Hill-Valley	Mean	47.57	48.14	22.71	41.71	34.71	44.95	35.57	48.24	33.76	13.29	17.00	17.52
	FRR	52.43%	51.86%	77.29%	58.29%	65.29%	55.05%	64.43%	51.76%	66.24%	86.71%	83.00%	82.48%
	Best	7	7	1	7	1	7	7	7	1	1	1	4
	Rank	10	11	4	8	6	9	7	12	5	**1**	2	3
Amazon	Mean	4764.62	4775.81	1006.19	2088.90	1557.29	4671.29	3570.14	4756.48	3878.19	476.95	2031.29	701.86
	FRR	52.35%	52.24%	89.94%	79.11%	84.43%	53.29%	64.30%	52.44%	61.22%	95.23%	79.69%	92.98%
	Best	7	7	7	7	7	7	7	7	7	7	7	7
	Rank	11	12	3	6	4	9	7	10	8	**1**	5	2
Arcene	Mean	4782.90	4761.71	510.71	2198.00	748.05	4602.67	3748.95	4766.38	687.90	965.38	495.24	429.05
	FRR	52.17%	52.38%	94.89%	78.02%	92.52%	53.97%	62.51%	52.34%	93.12%	90.35%	95.05%	95.71%
	Best	7	7	7	7	7	7	7	7	7	5	7	7
	Rank	12	10	3	7	5	9	8	11	4	6	2	**1**
DLBCL	Mean	2604.57	2598.86	1110.14	1435.19	645.71	2607.62	2353.67	2606.71	2198.67	754.71	239.71	208.05
	FRR	52.38%	52.48%	79.70%	73.76%	88.19%	52.32%	56.96%	52.34%	59.80%	86.20%	95.62%	96.20%
	Best	7	7	7	7	6	7	7	7	7	7	7	7
	Rank	10	9	5	6	3	12	8	11	7	4	2	**1**
Micro Mass	Mean	623.62	613.24	741.81	597.14	666.90	633.76	626.95	626.19	863.52	684.33	580.38	603.00
	FRR	52.03%	52.83%	42.94%	54.07%	48.70%	51.25%	51.77%	51.83%	33.58%	47.36%	55.36%	53.62%
	Best	7	7	7	7	7	7	7	7	7	7	7	7
	Rank	5	4	11	2	9	8	7	6	12	10	**1**	3
Leukemia	Mean	3389.52	3380.57	185.95	1205.19	190.76	3405.24	2324.95	3387.76	1266.38	112.19	136.19	141.76
	FRR	52.45%	52.58%	97.39%	83.09%	97.32%	52.23%	67.39%	52.48%	82.24%	98.43%	98.09%	98.01%
	Best	7	7	1	7	5	7	7	7	7	5	7	7
	Rank	11	9	4	6	5	12	8	10	7	**1**	2	3
Prostate	Mean	5976.48	6004.67	419.90	1335.38	600.38	6009.52	4978.00	5980.95	853.52	554.90	324.86	284.62
	FRR	52.57%	52.34%	96.67%	89.40%	95.24%	52.31%	60.49%	52.53%	93.23%	95.60%	97.42%	97.74%
	Best	7	7	7	7	7	7	7	7	7	7	7	1
	Rank	9	11	3	7	5	12	8	10	6	4	2	**1**
Colon	Mean	955.19	958.43	118.67	455.24	166.05	947.71	894.81	963.14	459.48	107.05	55.33	86.71
	FRR	52.24%	52.08%	94.07%	77.24%	91.70%	52.61%	55.26%	51.84%	77.03%	94.65%	97.23%	95.66%
	Best	7	7	7	7	3	7	7	7	7	3	1	7
	Rank	10	11	4	6	5	9	8	12	7	3	**1**	2
Average rank		7.8	7.9	5.6	5.2	5.0	7.9	7.1	8.1	7.6	6.6	4.6	**4.4**

**Table 6:** Comparison results in terms of *F*1-*score*.

		BABC	BBA	BCOA	BCPO	BHHO	BKOA	BNOA	BPSO	BSSA	BWOA	BWO	BGEPWO
Wine	Mean	0.9084	0.9338	0.9337	0.9338	0.9153	0.9471	0.9590	0.9365	0.9489	0.9409	0.9551	0.9608
	Rank	12	9	10	8	11	5	2	7	4	6	3	**1**
Sonar	Mean	0.7618	0.7851	0.7669	0.7833	0.7756	0.7735	0.7788	0.7721	0.7611	0.7692	0.7822	0.8054
	Rank	11	2	10	3	6	7	5	8	12	9	4	**1**
Breast Cancer	Mean	0.9378	0.9342	0.9410	0.9411	0.9397	0.9424	0.9362	0.9351	0.9393	0.9421	0.9406	0.9453
	Rank	9	12	5	4	7	2	10	11	8	3	6	**1**
Heart	Mean	0.8420	0.8549	0.8265	0.8173	0.8327	0.8260	0.8347	0.8212	0.8301	0.8498	0.8738	0.8799
	Rank	5	3	9	12	7	10	6	11	8	4	2	**1**
Dermatology	Mean	0.8975	0.9289	0.9508	0.9539	0.9570	0.9456	0.9393	0.9520	0.9469	0.9568	0.9602	0.9603
	Rank	12	11	7	5	3	9	10	6	8	4	2	**1**
Arrhythmia	Mean	0.1817	0.1962	0.1966	0.2576	0.2081	0.1853	0.1816	0.1706	0.2310	0.2340	0.2477	0.2727
	Rank	10	8	7	2	6	9	11	12	5	4	3	**1**
Lymphography	Mean	0.8572	0.9000	0.9160	0.9028	0.8849	0.8561	0.8728	0.8938	0.9103	0.8730	0.8704	0.8793
	Rank	11	4	**1**	3	6	12	9	5	2	8	10	7
SPECT	Mean	0.4567	0.2460	0.3743	0.4743	0.3215	0.4907	0.4759	0.4701	0.3025	0.4965	0.5001	0.5062
	Rank	8	12	9	6	10	4	5	7	11	3	2	**1**
Zoo	Mean	0.6850	0.6850	0.7602	0.8066	0.7503	0.7792	0.7719	0.7813	0.7569	0.8016	0.7794	0.7737
	Rank	12	11	8	**1**	10	5	7	3	9	2	4	6
Semeion	Mean	0.8864	0.8766	0.8953	0.8915	0.8919	0.8890	0.8940	0.8984	0.8779	0.8984	0.8949	0.8984
	Rank	10	12	4	8	7	9	6	1	11	3	5	**1**
Isolet	Mean	0.8688	0.8487	0.8610	0.8715	0.8636	0.8581	0.8664	0.8654	0.8571	0.8618	0.8606	0.8664
	Rank	2	12	8	**1**	6	10	3	5	11	7	9	3
Ionosphere	Mean	0.7144	0.7357	0.7653	0.7511	0.7518	0.7511	0.7540	0.7443	0.7735	0.7788	0.7775	0.8126
	Rank	12	11	5	8	7	9	6	10	4	2	3	**1**
Musk	Mean	0.8105	0.8233	0.8185	0.8421	0.8220	0.7996	0.8239	0.8396	0.8313	0.8232	0.8210	0.8341
	Rank	11	6	10	**1**	8	12	5	2	4	7	9	3
Hill-Valley	Mean	0.6257	0.6196	0.6248	0.6351	0.6287	0.6265	0.6300	0.6340	0.6224	0.6217	0.6080	0.6859
	Rank	7	11	8	2	5	6	4	3	9	10	12	**1**
Amazon	Mean	0.1657	0.1156	0.1264	0.1542	0.1261	0.1067	0.1135	0.1215	0.1234	0.1384	0.1322	0.1625
	Rank	**1**	10	6	3	7	12	11	9	8	4	5	2
Arcene	Mean	0.8185	0.7754	0.7987	0.8142	0.7963	0.7971	0.8005	0.8006	0.7965	0.8028	0.8168	0.8219
	Rank	2	12	8	4	11	9	7	6	10	5	3	**1**
DLBCL	Mean	0.8460	0.8416	0.8424	0.8587	0.8475	0.8336	0.8393	0.8368	0.8282	0.8461	0.8526	0.8633
	Rank	6	8	7	2	4	11	9	10	12	5	3	**1**
Micro Mass	Mean	0.4867	0.4794	0.4978	0.5018	0.4875	0.5038	0.4984	0.5075	0.4922	0.5094	0.5135	0.5213
	Rank	11	12	8	6	10	5	7	4	9	3	2	**1**
Leukemia	Mean	0.9154	0.8765	0.9025	0.9179	0.9095	0.9241	0.9149	0.9225	0.9092	0.8946	0.9244	0.9263
	Rank	6	12	10	5	8	3	7	4	9	11	2	**1**
Prostate	Mean	0.7487	0.7182	0.7582	0.7486	0.7322	0.7078	0.7169	0.6969	0.7265	0.7279	0.7571	0.7631
	Rank	4	9	2	5	6	11	10	12	8	7	3	**1**
Colon	Mean	0.8243	0.8253	0.8189	0.8173	0.8187	0.8152	0.8028	0.8097	0.8146	0.8258	0.8232	0.8298
	Rank	4	3	6	8	7	9	12	11	10	2	5	**1**
Average	rank	7.9	9.0	7.0	4.6	7.2	8.0	7.2	7.0	8.2	5.2	4.6	**1.8**

**Table 7:** Comparison in terms of the Wilcoxon rank-sum statistical test with 5%.

	BABC	BBA	BCOA	BCPO	BHHO	BKOA	BNOA	BPSO	BSSA	BWOA	BWO
Wine	3.59E-09+	3.40E-09+	0.00E+00=	0.00E+00=	0.00E+00=	0.00E+00=	1.86E-09+	3.42E-01=	1.86E-07+	1.98E-09+	1.79E-09+
Sonar	2.54E-06+	1.19E-07+	4.21E-08+	2.65E-06+	3.22E-08+	2.95E-08+	7.95E-08+	6.91E-07+	3.35E-08+	2.54E-05+	1.43E-03+
Breast Cancer	4.86E-05+	5.89E-07+	7.01E-09+	6.00E-09+	7.68E-09+	1.68E-04+	7.26E-09+	6.89E-09+	1.05E-08+	1.21E-01=	3.31E-02+
Heart	7.50E-07+	4.26E-07+	2.54E-04+	6.76E-02=	1.07E-03+	6.74E-05+	9.00E-05+	8.39E-02=	2.07E-04+	2.87E-03+	6.82E-01=
Dermatology	5.38E-07+	2.09E-08+	3.59E-08+	7.22E-01=	4.11E-07+	2.00E-02+	2.46E-06+	9.87E-03+	1.35E-07+	1.95E-03+	8.92E-01=
Arrhythmia	2.69E-07+	3.06E-08+	3.41E-08+	8.51E-03+	3.91E-07+	5.30E-01=	2.87E-08+	3.57E-08+	1.73E-01=	8.92E-07+	9.09E-05+
Lymphography	7.12E-07+	1.02E-07+	4.06E-08+	9.03E-08+	3.09E-08+	2.62E-06+	2.02E-08+	2.20E-08+	2.70E-08+	3.20E-01=	8.60E-02=
SPECT	3.61E-06+	6.32E-06+	1.22E-08+	1.60E-08+	1.39E-08+	1.51E-06+	1.49E-08+	1.43E-08+	9.44E-09+	5.76E-01=	1.70E-08+
Zoo	1.10E-04+	5.04E-06+	1.00E+00-	1.00E+00-	1.00E+00-	9.78E-01-	1.00E+00-	1.00E+00-	1.00E+00-	2.24E-01=	8.16E-01=
Semeion	8.19E-04+	4.39E-08+	6.71E-02=	3.02E-08+	4.70E-03+	3.22E-08+	3.24E-08+	2.10E-04+	3.30E-08+	3.84E-06+	7.05E-03+
Isolet	8.76E-09+	2.28E-09+	2.12E-09+	3.67E-09+	2.25E-09+	2.16E-09+	2.24E-09+	7.26E-08+	3.06E-09+	2.24E-09+	5.57E-08+
Ionosphere	1.30E-05+	2.75E-08+	6.25E-04+	6.57E-01=	1.15E-04+	2.51E-08+	7.26E-08+	7.72E-06+	6.90E-06+	2.88E-07+	4.14E-05+
Musk	1.70E-05+	2.77E-08+	2.98E-08+	6.99E-07+	2.83E-08+	2.88E-08+	2.14E-08+	9.96E-03+	2.90E-08+	6.18E-07+	7.62E-01=
Hill-Valley	3.08E-08+	2.99E-08+	2.55E-08+	2.32E-08+	2.53E-08+	2.77E-08+	2.77E-08+	2.68E-08+	3.00E-08+	2.86E-08+	2.93E-08+
Amazon	6.11E-03+	2.53E-06+	6.08E-08+	7.28E-06+	2.74E-07+	3.30E-08+	3.74E-08+	3.89E-07+	2.79E-04+	1.02E-06+	5.34E-01=
Arcene	3.96E-06+	3.21E-07+	2.99E-06+	8.46E-06+	3.56E-07+	2.60E-08+	2.35E-08+	3.52E-08+	6.03E-05+	3.90E-05+	2.65E-01=
DLBCL	1.29E-02+	1.09E-01=	2.53E-05+	7.00E-05+	3.69E-03+	2.48E-06+	4.15E-09+	1.55E-09+	4.00E-07+	1.46E-03+	6.19E-02=
Micro Mass	1.11E-06+	5.05E-08+	3.08E-08+	1.95E-02+	9.85E-08+	3.07E-08+	3.04E-08+	1.00E+00-	1.91E-04+	5.37E-07+	2.55E-02+
Leukemia	4.39E-04+	1.08E-06+	8.61E-06+	1.43E-08+	3.30E-06+	3.58E-09+	1.27E-08+	6.00E-09+	1.70E-07+	1.20E-05+	1.08E-02+
Prostate	5.12E-01=	2.23E-05+	3.96E-04+	1.95E-02+	6.88E-05+	2.46E-09+	1.90E-08+	2.40E-08+	8.14E-04+	1.02E-02+	3.33E-01=
Colon	4.14E-01=	4.25E-07+	1.37E-05+	5.35E-07+	5.24E-06+	5.22E-03+	1.06E-08+	4.89E-09+	9.30E-07+	1.82E-04+	1.03E-02+
+/=/−	19/2/0	20/1/0	18/2/1	16/4/1	19/1/1	18/2/1	20/0/1	17/2/2	19/1/1	17/4/0	12/9/0

## Data Availability

Data are contained within the article.
